# Microgravity and Cell Adherence

**DOI:** 10.3390/ijms21062214

**Published:** 2020-03-23

**Authors:** Johann Bauer

**Affiliations:** Max Planck Institute f. Biochemistry, Am Klopferspitz 18, D-82152 Martinsried, Germany; jbauer@biochem.mpg.de; Tel.: +49-89-8578-3803

Cell adhesion is an inevitable precondition for enabling cells to assemble into three-dimensional tissues [1]. Different cell adhesion mechanisms are responsible for determining the overall architecture of a tissue. Central elements of the cell adhesion systems are transmembrane proteins, which include various integrins, cadherins, immunoglobulins, selectins and proteoglycans. At the extracellular surface, they are most often glycosylated and form receptors, which recognize receptors of surrounding cells or proteins of the extracellular matrix [2]. At the cytoplasmic surface, these transmembrane proteins are associated with a number of peripheral membrane proteins and cytoplasmic proteins, which establish a dynamic link between the adhesion receptors and the cytoskeleton and quite often initiate signals towards the cell nucleus [3,4].

Cell adhesion is a dynamic process, which stays under the influence of physiological, developmental, and pathological factors and exerts a number of effects on the life of cells [1,5,6]. In recent years, it became evident that even gravity generates effects affecting cell adhesion [7]. It was recognized that the adherence of human and animal tissue cells is changed when they are exposed to microgravity. Changes in adherence became especially apparent when scaffold-free organoid formation was investigated, which included the cells’ detachment from the surface of the culture flasks followed by three-dimensional aggregation. Investigating this process, distinct proteins and genes coding for them have been identified, which contribute either to a cell’s adherence to a substratum or to contact with neighboring cells [8]. The type, force and effects of adherence are regulated by external and internal signals. External signals are caused by features of the surface to which the cells adhere [9]. Internal signals are generated by the influence of gravity on protein complexes [10].

In this Special Issue, eight articles [11,12,13,14,15,16,17,18] and one review [19] have been published. They cover various aspects of changes in cellular adhesion behavior under microgravity and indicate related changes in cell vitality and gene expression or protein accumulation. These were detected in cells after exposure to real [16,17] or simulated microgravity in vitro [11,12,13,14] and in vivo [15].

A well-known effect of exposing cells to microgravity is the induction of apoptosis. In detail, however, the microgravity-dependent induction of apoptosis is not completely understood. Sokolovskaya et al. described an investigation of Jurkat cells in comparison to mutant Jurkat/A4 cells, which had acquired multidrug resistance [12]. They found a higher sensibility of the mutant Jurkat/A4 cells for the induction of apoptosis by simulated microgravity. In these cells, the intercellular adhesion molecule 3 was reduced when they were cultured on a random positioning machine (RPM) for 96 h. Also, in lung cancer cells, incubation on an RPM induced apoptosis within 24 h [14]. Interestingly, the increase in the rate of apoptosis was much higher in those cells which formed spheroids under simulated microgravity than in those which remained adherent. The lower enhancement of apoptosis in adherent cells was associated with the expression of the tumor suppressor genes *TP53, CDKN2A, PTEN*, and *RB1*. The onset of apoptosis appears to take some time, because, in a parabolic flight mission, signs of apoptosis could not be observed within about two hours of stressing the cells by hypergravity, hypogravity and vibration, as after this time, the mRNA levels of PRKCA, RAF1, BAX were not changed and cleaved caspase-3 was not detectable. However, mRNA levels of ICAM1, CD44 and ERK1 were down-regulated after the first parabola and a delayed up-regulation of FAK1 after the last 31st parabola was observed [16]. This suggested that genes of adhesion-related proteins responded to microgravity, before apoptosis became obvious. A very fast induction of structural changes of adhesion and cytoskeletal proteins was also proved by a rocket flight, which exposed the cells to real microgravity for six minutes. At the end of this flight, structural alterations of tubulin and F-actin were observed by life-cell microscopy [17]. Although very quickly induced, cytoskeletal changes persisted in Ewing’s sarcoma cells at least over 24 h culturing on the RPM [13]. The changes were more obvious in spheroid cells than in adherent cells, which both had been cultured on the RPM. Only in spheroid cells, *CXCR4* and *CD44* expression were significantly enhanced, while CD44 protein decreased in spheroids and adherent cells. However, the inhibition of CXCR4 did not change spheroid count or structure. While the changes described above occurred in Ewing’s Sarcoma cells, *CAV1* was up-regulated.

This is further proof of the important role which CAV1 plays in the regulation of cell adherence under microgravity. Caveolin-1 is a scaffold protein that is anchored in the lipid bilayer and has binding sites for various proteins. It responds to changes in gravity and has influence on spheroid formation [20]. The observations on the sarcoma cells [13] fits to the knowledge obtained by a semantic approach, which revealed two enzymes whose activity depends on a binding to caveolin-1. These enzymes either sialylate (ST6GAL1) or de-sialylate (NEU1) the extracellular domains of receptor proteins, which recognize neighboring cells or ECM proteins. In this way they generate a status of sialylation, which has influence on binding activity of adhesion receptor proteins and regulates cell migration and metastasis in vivo [18]. A link between cell adhesion in vitro and metastasis in vivo was also proved by reviewing the behavior of human melanoma cells with different metastatic potential [19]. Different types of melanocyte showed different melanocyte–extracellular matrix interactions under simulated microgravity, while intracellular signals of cyclic guanosine-monophosphate (cGMP) were regulated differently.

The importance of the strength by which tissue cells bind to the underlay was further shown by Shuliang Shi et al. [11]. After culturing healthy human umbilical vein endothelial cells (HUVEC) or malignant human breast cancer cells in a rotating wall vessel (RWVS), the authors observed that adhesion strength of the cells to the underlay decreased so that more cells could be exfoliated under equal treatment from slips mounted on the RWVS than from equal slips used for culturing these cells at 1g normal gravity. Together with the decrease of adhesion strength integrin β1, paxillin and e-cadherin proteins were reduced, while the EMT (epithelial-mesenchymal transition) transcription factors Snail, Twist, and ZEB1 were up-regulated. Hence, the paper strongly suggests a link between the adhesion strength to the extracellular matrix via integrin β1 or to neighboring cells via e-cadherin and an induction of EMT. Paxillin appears to be an important mediator between the adhesion receptors and the EMT. When cells are exposed to simulated microgravity, paxillin regulates the stability of adhesion complexes depending on the presence of ASAP1 or Bcar1 [21].

The strength of cell adherence appears to play a role in wound healing also. This can be suggested upon the report of Cialdai et al. [15]. They had investigated the influence of simulated microgravity on wound healing in vivo [15]. The authors observed a delay and structural alterations in the repair tissue, when a leech with a wound was kept on an RPM. This impairment of wound healing appeared to be due to an impairment in fibroblast migration to the wound site under simulated microgravity. It could be counteracted by adding platelet rich plasma (PRP), but a defined factor causing this effect remains to be determined,

Overall, this Special Issue provides new data and novel insights into the complexity of cell adherence and its alteration upon omitting gravity. Knowledge is provided about the causes and consequences of microgravity-caused reduction in cell adhesion strength and their relationships to tumor growth and metastasis as well as to wound healing. Further investigation of the numerous proteins and genes linked to the cell adhesion behavior under microgravity will surely show targets for curing cancer or supporting wound healing.

## Abbreviations

ASAP1 PTK2/FAK1	Arf-GAP with SH3 domain, ANK repeat and PH domain-containing protein 1 Focal adhesion kinase 1
Bcar1	Breast cancer anti-estrogen resistance protein 1
CAV1	Caveolin-1
CD44	CD44 antigen
CDKN2A	Cyclin-dependent kinase inhibitor 2A
CXCR4	C-X-C chemokine receptor type 4
ECM	Extracellular matrix
EMT	epithelial-mesenchymal transition
ERK1	Mitogen-activated protein kinase 3
HUVEC cGMP	Umbelical vein endothelial cells Cyclic guanosine monophosphate
ICAM1	Intercellular adhesion molecule 1
NEU1	Sialidase 1 /Neuraminidase 1
PRKCA	Protein kinase C alpha type
PRP	Platelet rich plasma
PTEN	Phosphatidylinositol 3,4,5-triphossphate 3-phosphatase and dual-specific protein phosphatase PTEN
RAF1	RAF proto-oncogene serin/threonine-protein kinase
RB1	Retinoblastoma-associated protein
RPM	Random positioning machine
RWVS	Rotating Wall Vessel
Snail,	Zinc finger protein SNAI1
ST6GAL1	Beta-galactoside alpha-2,6-sialyltransferase 1
TP53	Cellular tumor antigen p53
Twist	Twist related protein
ZEB1	Zinc finger E-box-binding homeobox 1
